# Method of Microglial DNA-RNA Purification from a Single Brain of an Adult Mouse

**DOI:** 10.3390/mps4040086

**Published:** 2021-12-02

**Authors:** Md. Obayed Raihan, Brett A. McGregor, Nathan A. Velaris, Afrina Brishti, Junguk Hur, James E. Porter

**Affiliations:** Department of Biomedical Sciences, University of North Dakota School of Medicine and Health Sciences, Grand Forks, ND 58202, USA; mdobayed.raihan@und.edu (M.O.R.); brett.mcgregor.3@und.edu (B.A.M.); nathan.velaris@und.edu (N.A.V.); afrina.brishti@und.edu (A.B.)

**Keywords:** microglia isolation, DNA/RNA extraction, column-based separation

## Abstract

Microglia, the resident brain immune effectors cells, show dynamic activation level changes for most neuropsychiatric diseases, reflecting their complex regulatory function and potential as a therapeutic target. Emerging single-cell molecular biology studies are used to investigate the genetic modification of individual cells to better understand complex gene regulatory pathways. Although multiple protocols for microglia isolation from adult mice are available, it is always challenging to get sufficient purified microglia from a single brain for simultaneous DNA and RNA extraction for subsequent downstream analysis. Moreover, for data comparison between treated and untreated groups, standardized cell isolation techniques are essential to decrease variability. Here, we present a combined method of microglia isolation from a single adult mouse brain, using a magnetic bead-based column separation technique, and a column-based extraction of purified DNA-RNA from the isolated microglia for downstream application. Our current method provides step-by-step instructions accompanied by visual explanations of important steps for isolating DNA-RNA simultaneously from a highly purified microglia population.

## 1. Introduction

Microglia, the brain’s resident immune cells, account for 5–12% of total cell population in the mouse brain and play a crucial role in adult brain neurogenesis, neuroinflammation, and overall brain homeostasis [1]. This multifunctional brain cell vastly depends on environmental stimuli to maintain its homeostatic phenotype [2] and immediately adjusts its functional profile based on the physiological or pathophysiological needs of the individual. Microglia mediate crosstalk between the brain and immune system, participating in the pathogenesis of neurodegenerative disorders and protecting against invading pathogens [3,4]. Characterization of the inflammatory profiles associated with crucial epigenetically regulated genes and connected signaling pathways in microglia are therefore significant in identifying potential therapeutic targets contributing to the pre-clinical progression of neurodegenerative disorders. Crosstalk between microglia and immune cells like major histocompatibility complex class II (MHC class II) and CD74 have recently been studied in multiple sclerosis (MS) pathology [5], which has increased our understanding of chronic inflammation observed in MS. Microglial genetic defects in neurodegenerative disorders may have a comprehensive impact on disease-specific pathology. Modulation of microglial function by gene editing on the whole brain or specific brain regions could hold promise for therapeutic targets in many neurodegenerative and neuropsychiatric diseases.

Accumulating evidence suggests that differences in DNA isolation methods could have a significant impact on downstream applications, especially relative mitochondrial DNA (mtDNA) abundance in whole-genome sequencing [6,7]. Similarly, differences in RNA isolation methods severely affect relative transcript abundance in RNA sequencing analysis [8,9]. Despite having a number of microglia isolation protocols available [10,11,12,13,14,15,16], it is always challenging to isolate purified microglia from a single mouse brain as it often results in low yields. Therefore, for reliable data comparisons, it is a prerequisite to adopt combined standardized DNA and RNA isolation methods along with a standard microglia purification method with the same conditions applied within all independent experimental batches.

CD11b is the most commonly used microglia selection marker from the central nervous system, as it is expressed by both resting and activated microglia like Iba1 [17]. Although the cell surface antigen CD45 is also expressed by both resting and activated microglia, the expression varies depending on microglial phagocytic capacity under pathological conditions [18]. TMEM119 is another cell-surface protein recently used as a specific microglial marker that can distinguish brain microglia from invading monocytes in disease conditions. However, TMEM119 expression is absent in immature microglia and the number of infiltrating monocytes under pathological conditions is very low compared to brain resident microglia [19].

Here, we present a combined and step-wise method adapted from previously described protocols [10,11,12,13,14,15,16,20,21] to isolate intact DNA and RNA from a highly pure microglial population from one single mouse brain using CD11b as a microglia selection marker. Isolated DNA and RNA using this method could be suitable for downstream molecular biology applications, including methylated DNA and transcriptome analysis designed for understanding genetic profiles.

## 2. Experimental Design

### 2.1. Materials

Table 1 shows the detailed information of all the chemicals, kits, and supplies.

### 2.2. Equipment

Table 2 shows the detailed information of the equipments.

### 2.3. Freshly Prepared Solution (to Be Made Fresh Immediately before Experiment)

Table 3 shows the detailed information of the freshly prepared solutions.

## 3. Procedure

### 3.1. Part 1: Brain Tissue Dissociation (100 min/up to 6 Mice)

(Note: Animal surgery room should be cleaned and disinfected; all steps should be performed using aseptic technique under class II biological hood.)

1. Adult mice (>4 weeks old) euthanatize with isoflurane/CO_2_/Pentobarbital.

(Note: Ensure euthanasia by paw pinching. For multiple mice, euthanize one after another for single hand.)

2. Perfuse the mice transcardially (through left ventricle) with 10–15 mL of ice-cold 1 × DPBS with calcium and magnesium before harvesting the brain.

(Note: Transcardiac perfusion eliminates circulatory CD11b positive cells from the brain; Therefore, continue perfusion till the last heartbeat for complete elimination.)

3. Cut the skull upward along the sagittal suture starting from the brain stem, peel away two halves of the skull and scoop out the whole brain.

(Note: If transportation is needed, transfer the whole brain in a 15/50 mL sterile falcon tube containing ice-cold 1 × DPBS with calcium and magnesium placed in an ice box.)

**Figure 1:**
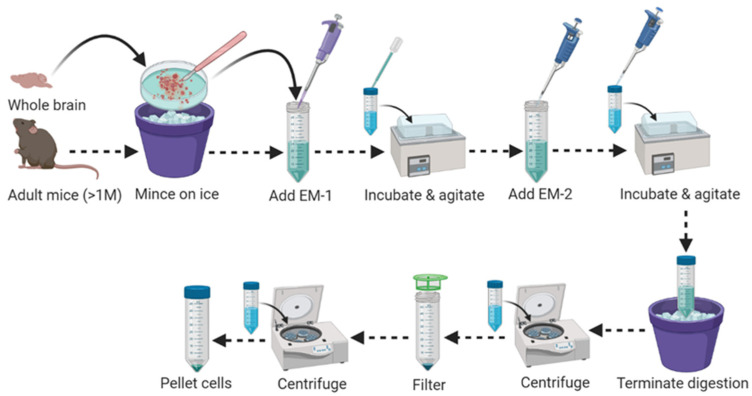
Schematic workflow for adult mouse brain tissue dissociation.

4. Place the brain segment into a sterile Petri dish on ice and wash 2–3 times with 1 mL of ice-cold 1 × HBSS without calcium and magnesium.
5. Apply a sterile, sharp knife and forceps to mince the whole brain into multiple (10–15) slices on the same Petri dish, containing 1 mL of ice-cold 1×HBSS without calcium and magnesium.
6. Transfer all minced brain slices (for a single brain) into a 15 mL Falcon tube containing 1950 μL MACS Enzyme Mix-1/EM-1 (1900 µL Buffer Z + 50 µL Enzyme P) pre-warmed at 37 °C for 5 min to aid in tissue dissociation step in Figure 1.
7. Incubate the sample mixture in a water bath at 37 °C for 15 min with continuous shaking at a speed of 50 revolutions per minute (r.p.m.).
8. At the end of incubation, agitate the aggregated tissue slices using a flame polished glass Pasteur pipette to make a single cell suspension (pipet up and down approximately 20 times or until all tissues move freely up into the Pasteur pipette).

(Note: Do not vortex or invert the tube as tissue slices may stick in the lid or on the walls, resulting in incomplete digestion.)

9. Add 30 µL of MACS Enzyme mix-2/EM-2 (20 µL Buffer Y + 10 µL Enzyme A) into the agitated sample mix. Mix by pipetting; do not vortex or invert the tube.
10. Incubate the sample mix in a water bath at 37 °C for 10 min with continuous shaking at a speed of 50 r.p.m.
11. At the end of incubation, agitate the sample mixture 20–25 times using a 1 mL pipette.
12. Add DNase I (5 U/mL) into the agitated sample mix. Mix by pipetting; do not vortex or invert the tube.
13. Incubate the sample mix in a water bath at 37 °C for 10 min with continuous shaking at a speed of 50 r.p.m.
14. At the end of incubation, to completely stop the digestion reaction, dilute the tissue sample with 2 mL of ice-cold 1 × HBSS without calcium and magnesium and incubate on ice for 5 min.
15. Centrifuge tissue samples quickly at 800–1000× *g* for 10–15 min at room temperature (20–25 °C), decant the supernatant, and collect the cell pellet.
16. Resuspend cell pellets in 2 mL of ice-cold 1 × HBSS without calcium and magnesium.
17. Apply resuspended cell suspension directly into the middle of the pre-moisten MACS SmartStrainer (70 μm) placed on a sterile 50 mL Falcon tube.

(Note: Pre-wet/moisten the MACS SmartStrainer (70 μm) with 1–2 mL of ice-cold 1 × HBSS with calcium and magnesium and discard the flow-through. Improper or incomplete pre-wetting of the Strainer filter can cause cell stacking and sticking around the upper side of the Falcon tube surrounding the filter and increase cell loss.)

18. Apply 7 mL of ice-cold 1 × HBSS with calcium and magnesium on the MACS SmartStrainer (70 μm). Rinse the old Falcon tube carefully by pipetting up and down with 1 mL 1 × HBSS with calcium and magnesium, and add it directly to the MACS SmartStrainer to prevent any cell loss.
19. Discard the MACS SmartStrainer (70 μm) and centrifuge cell suspension at 600–700× *g* for 10 min at 4–8 °C. Carefully remove the supernatant by vacuum aspiration or by pipetting.

### 3.2. Part 2: Debris Removal (40 min)

**Figure 2:**
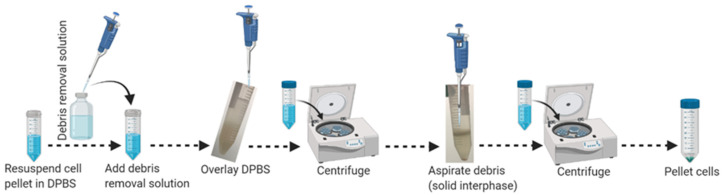
Schematic workflow for debris removal from adult brain single-cell suspension.

20. Resuspend cell pellets gently by pipetting with the appropriate volume (3100 μL/single brain) of ice-cold 1 × DPBS, without calcium and magnesium, and transfer cell suspension into a new sterile 15 mL conical tube. Do not vortex.
21. Add the appropriate volume (900 μL/single brain) of cold MACS debris removal solution into the resuspended sample and mix gently by pipetting 10–12 times as outlined in Figure 2.
22. Overlay 4 mL of ice-cold 1 × DPBS without calcium and magnesium very gently above the cell suspension to make a transparent gradient.

(Note: Start overlaying by holding the tube at a 45° angle and slowly bring the tube back to a vertical position as more DPBS is added on top of the layer of debris removal solution. Pipet very slowly to ensure that the two phases are not mixed.)

23. Centrifuge at 1000× *g* for 10 min at 4 °C with maximum acceleration and full brake.

(Note: After centrifugation, remove the tube from the rotor carefully so as not to agitate the three different phases (top liquid, middle solid debris, and bottom liquid). Supplemental Figure S1 shows the pre- and post-centrifugation gradients.)

24. Aspirate the two top phases (top liquid and solid interphase) completely and discard them.

(Note: Work quickly, as the solid debris interphase gradually settles down over time. Gently insert the pipette near the side of the solid interphase, then let the pipette tip touch the solid interphase and aspirate the solid waste gently, followed by the top liquid phase removal.)

25. Fill the tube with the appropriate volume (~11 mL) of ice-cold 1 × DPBS without calcium and magnesium to a final volume of 14 mL.
26. Gently invert the tube three (3×) times. Do not vortex!
27. Centrifuge the tube at 1000× *g* for 10 min at 4 °C with maximum acceleration and full brake.

# During centrifugation, make a fresh PB buffer (e.g., 1 × DPBS without calcium and magnesium with 0.5 × BSA and add 50 μL BSA into 9950 μL of 1 × DPBS to make 10 mL of PB buffer). Before use, sterilize the filter first with PB buffer and then degas by vacuuming at room temperature, as gas may clog the separation column and affect the quality of separation. Keep the buffer cold at 2–8 °C. This is a preparatory procedure for Step 40 in Section 3.5., Part 5, to streamline the protocol and minimize wasted time.

# Prepare 1 × Red Blood Cell (RBC) removal solution for a single mouse brain by mixing 900 μL of sterile ddH_2_O with 100 μL of MACS 10 × RBC removal stock solution. This solution is for Step 28 in Section 3.3., Part 3.

### 3.3. Part 3: Red Blood Cell (RBC) Removal (30 min)

**Figure 3:**
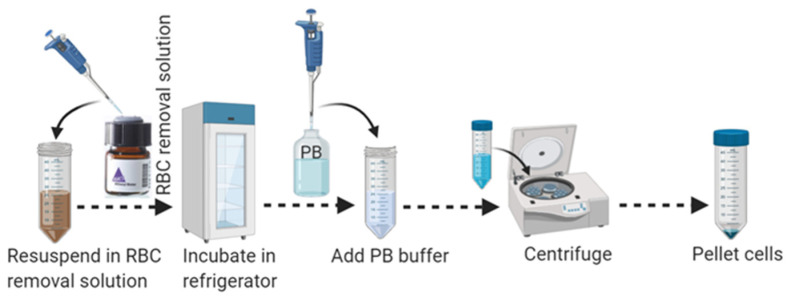
Schematic workflow for RBC removal from adult brain cell suspension.

28. Aspirate supernatant completely and resuspend cell pellets carefully in 1 mL of cold 1 × RBC removal solution by pipetting up and down 10–15 times with a 1 mL pipette. Do not vortex.
29. Incubate the cell mixture for 10 min in the refrigerator at 2–8 °C.
30. Add 10 mL of ice-cold PB buffer to the cell mixture to wash out the RBC removal solution.
31. Centrifuge at 400× *g* for 10 min at 4 °C. Aspirate supernatant completely to eliminate RBC as shown in Figure 3.

### 3.4. Part 4: Magnetic Labeling (45 min)

32. Resuspend cell pellets (from the single mouse brain) in 150 µL of ice-cold PB buffer by gently pipetting up and down. Transfer resuspended cell suspension to a sterile 1.5 mL microcentrifuge tube. Do not vortex cell suspension.

(Note: Keep cells cold and use pre-cooled solutions during magnetic labeling to prevent any capping of antibodies on the cell surface that may lead to non-specific cell labeling.)

**Figure 4:**
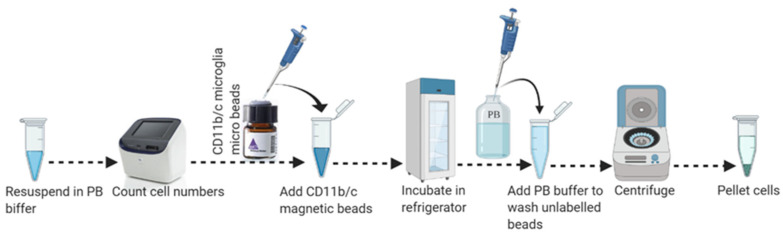
Schematic workflow for magnetic labeling of CD11b-positive microglial cells from adult brain single-cell suspension.

33. Transfer 15 µL of the cell suspension to a new tube and proceed to cell count. Use 135 μL of cell suspension (for up to 5 × 10^7^ total cells/single mouse brain) for magnetic labeling as shown in Figure 4.
34. Add 15 µL of MACS CD11b magnetic (Microglia) micro-beads (human and mouse) into the 135 µL of cold cell suspension and mix by pipetting gently up and down 10–15 times with 200 µL pipette.

(Note: Protect the magnetic beads and cell beads mixture from light. For optimal labeling, it is important to adjust the volume of PB buffer/beads ratio, for instance, with ≤1 × 10^7^ total cells in 90 μL buffer, add 10 μL microglia micro-beads, and with ≥1 × 10^8^ total cells in 180 μL buffer, add 20 μL microglia micro-beads.)

35. Place the tube (cell-beads mixture) in an End Over End shaker, keep the mix protected from light, and incubate for 15 min in the refrigerator at 2−8 °C.

(Note: At this stage, turn on the microcentrifuge machine and cool down the temperature to 4 °C for the next centrifugation steps.)

36. Add 1–2 mL of cold PB buffer into the cell-beads mixture per 10^8^ cells and centrifuge at 300–350× *g* for 10 min at 4 °C to wash out the unlabeled beads. Aspirate supernatant completely.
37. Resuspend magnetically labeled cells (up to 5 × 10^7^ cells/single mice brain) carefully in 500 µL of cold PB buffer and remove any bubbles if formed during resuspension.

(Note: For higher cell numbers, scale up the PB buffer volume proportionately.)

38. Transfer 40 µL of the cell suspension to another sterile 1.5 mL tube from the 500 µL cells for later flow cytometric analysis (this cell fraction is designated as the total cells fraction or original cell fraction for FACS compensation analysis).

### 3.5. Part 5: Magnetic Separation of CD11b Positive Cells (35 min)

**Figure 5:**
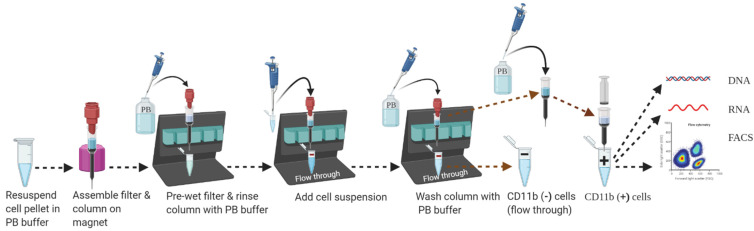
Schematic workflow for magnetic separation of CD11b positive cells from adult brain cells suspension.

39. Assemble miniMACS MS column (label up to1 × 10⁷ cells from up to 1 × 10^8^ total cells) inside the miniMACS magnet attached to a suitable MACS separator as shown in Figure 5. Place an open 1.5 mL collection tube below the column to collect flow-through.
40. Place MACS pre-separation filter (30 µm) on top of the column to remove any cell clumps which may clog the column.

(Note: Pre-wet/moisten the pre-separation filter with PB buffer before use. For optimal separation, it is important to obtain a single-cell suspension before magnetic separation. Microglia has its territory about 15–30 µm wide, and the miniMACS MS columns are not suitable for particle sizes greater than 30 µm.)

41. Apply 500 µL degassed PB buffer on the middle of the pre-separation filter and let the buffer run through the column (for LS column, apply 3 mL of buffer) to activate the column. Discard the flow-through and replace the collection tube with a new one.

(Note: Add CD11b-magnetic labeled cell suspension immediately after filling the degassed PB buffer to avoid the formation of air bubbles caused by warming up. Do not freeze or store column after filling.)

42. Apply 460 μL of CD11b-magnetic labeled cell suspension on a pre-separation filter placed on top of the column. Collect flow-through in a sterile 1.5 mL microcentrifuge tube labeled as negative fraction or CD11b (−) cell fraction.

(Note: To avoid cell loss, add cell suspension on the middle of the filter.)

43. Wash the miniMACS MS column by adding 500 µL of PB buffer on the pre-separation filter 3 times (for the LS column, use 2 mL of PB buffer 3 times). Collect unlabeled cells that pass through and combine with the flow-through from Step 42. This is the CD11b (−) cell fraction or negative fraction.

(Note: Perform the individual washing step by adding PB buffer as soon as the column reservoir becomes almost empty. Generally, it takes around 1 min for 500 μL of PB buffer to follow through the MS column; however, depending on the washing buffer composition or viscosity of the flow-through, time may vary.)

44. Remove the miniMACS MS column gently from the magnetic field and place it on a suitable (a sterile 1.5 mL microcentrifuge tube for the MS column and a 5 mL tube for the LS column) collection tube held on ice. Place the flow-through or CD11b (−) cell fraction collection tube immediately on ice.
45. Pipet an appropriate amount of PB buffer (for the miniMACS MS column: 1 mL and for the LS column: 5 mL; for the MS column, first add 200 uL of PB buffer to pass without pressure, then add 800 uL of PB buffer to flush out with a plunger) into the column reservoir. Push the plunger firmly inside the column reservoir and immediately flush out the magnetically labeled cells in the collection tube. This is the target cell fraction (positive fraction, target fraction, CD11b (+) positive cell fraction, or microglia population).

(Note: Although optional, it is recommended to add another 200 μL of PB buffer for the miniMACS MS column and 500 μL for the LS column into the column reservoir. Immediately flush out and combine the fraction with the positive fraction from Step 45 to prevent any cell loss.)

46. To increase the purity of isolated microglial cells, it is recommended to enrich the positive fraction over a second miniMACS MS or LS column and repeat the magnetic separation procedure from Step 41 to Step 45, applying a new separation column.

(Note: After the second column filtration, CD11b (+) cell fraction in the tube will look “transparent” and cells may not be visible with unaided eyes whereas CD11b (−) cells in the tube look “thick” due to the highly dense cell population.)

47. Pellet CD11b (+) cell fraction or microglia cells in the PB buffer by centrifugation at 300–400× *g* for 10 min at 4 °C.
48. Aspirate supernatant very carefully, leaving a meniscus of liquid (~50 μL) at the bottom of the tube (as CD11b+ cells pellet, they may not be visible to unaided eyes) to prevent any cell loss.
49. Resuspend cell pellet in 1 mL of 1 × DPBS, centrifuge at 300–400× *g* for 10 min at 4 °C, and aspirate supernatant carefully.
50. Loosen the cell pellets by flicking the microcentrifuge tube 2–3 times and resuspend cell pellets in media of choice for downstream applications.

(Note: (A) Add 350 μL of RLT plus buffer from the AllPrep DNA/RNA mini kit for downstream simultaneous DNA/RNA purification. (B) Add 500 μL of RIPA buffer containing protease inhibitor cocktail for downstream proteomic analysis. (C) Add 1 mL of cell culture medium (DMEM/F-12 with 10 × FBS and 1 × penicillin-streptomycin) to wash, centrifuge 300–400× *g* for 10 min at 4 °C, and resuspend in the cell culture medium for downstream cell culture and ex vivo analysis. (D) Add 1 mL of FACS stain buffer and resuspend the microglial cell pellets for downstream FACS analysis.)

### 3.6. Part 6: FACS Analysis (100 min)

(Before beginning;

A. Centrifuge newly received antibodies at high speed to remove “aggregated immunoglobulin”, a major source of nonspecific binding.
B. Prepare fresh buffers:

FACS staining buffer: Add 2 × FBS in 1 × DPBS without calcium and magnesium.

FACS buffer: Add 5 mM EDTA to FACS buffer to avoid cell aggregation.

C. Always stain on ice to prevent internalization of antigen-antibody complexes.
D. Keep stained sample protected from light and heat to avoid tandem degradation.
E. Always include a live/dead (viability dye) staining dye to discriminate live cells.
F. Titrate optimal antibody concentration to maximize the signals to noise ratio. High antibody concentration can increase non-specific binding whereas lower concentration can decrease signal intensity.
G. Carefully select the antibody conjugate. Fluorophores with the highest staining index (such as PE, APC) are best used for cells that have the lowest antigen expression or lower subsets, whereas dimmer fluorophores (PerCP, Alexa Fluor 405) are better suited for more highly expressed antigens or higher subsets.
H. The combined volume of the cell sample and antibody should not exceed 100 μL. High concentration or dilution may affect staining efficiency.
I. For multiple antigens staining, make an antibody cocktail with FACS stain buffer and then add equally to the designated cell suspension.)

**Figure 6:**
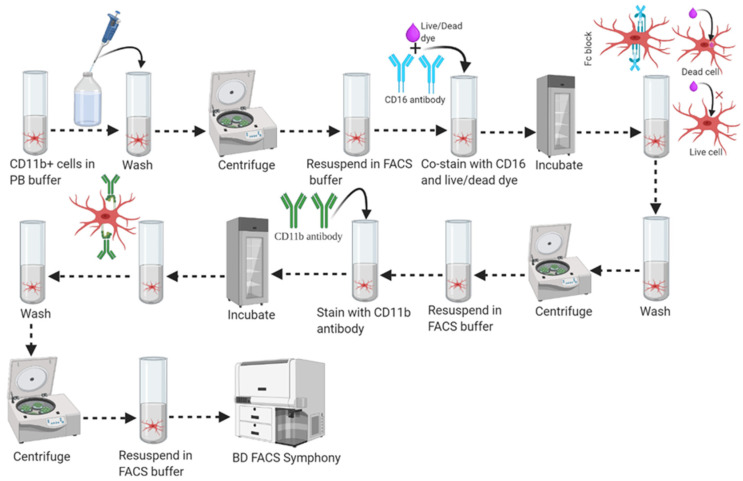
Schematic workflow for fluorescence staining strategy in FACS analysis.

1. Transfer 100 µL of CD11b (+) cell fraction (~1.0 mL stock) and CD11b (−) cell fraction (~1.8 mL stock from Step 46) into two different round-bottom FACS Falcon tubes as shown in Figure 6.
2. Distribute 40 µL of the “total cells fraction” or “original cell fraction” from Step #38 to another four (4) FACS Falcon tubes (individual tubes labeled as Unstained, CD11b-APC single stain, live/dead-violet 510 single stain, and all stain) and adjust each individual tube’s final volume to 100 μL with 90 μL of FACS stain buffer.

(Note: At this stage, take out the live/dead staining dye vial (Tonbo Bioscience, Ghost Dye, Violet #510) from the −20 °C freezer, keep it from light, and allow it to equilibrate to room temperature. Before use, quickly spin Ghost dye vial.)

3. Wash the cells once in 1 mL of FACS stain buffer and centrifuge at 350–400× *g* for 10 min at 20–25 °C. Resuspend cells in 90 μL of FACS stain buffer.

(Note: Resuspend up to 10^7^ cells per 100 μL of FACS stain buffer. For higher cell numbers, scale up the volume of all reagents accordingly, e.g., for 2 × 10^7^ cells, and double the volume of suspension buffer, antibody concentration, and washing buffer.)

4. Add 1 μL of live/dead staining dye (Tonbo Bioscience, Ghost Dye, Violet #510) to the cell suspension and vortex to mix immediately.
5. Incubate in 2–8 °C freezer for 30 min in the dark.
6. Wash cells once in 1 mL of FACS stain buffer and centrifuge at 350–400× *g* for 10 min at 20–25 °C. Resuspend cells in 100 μL of FACS stain buffer.
7. Add 1/2 μL (Tonbo Bioscience) of anti-mouse CD16/CD32 (Fc shield) antibody to the cell suspension and vortex to mix.

(Note: Fluorophore-conjugated antibodies may bind to cell surface Fc receptors and contribute to non-specific staining. It is therefore recommended to block Fc-receptors (anti-mouse CD16/CD32 monoclonal antibody) before intended surface receptor (CD11b) staining.)

8. Incubate cell suspension with CD16/CD32 antibody in a 2–8 °C freezer for 5 min in the dark.
9. Add 5 μL of anti-mouse and human CD11b antibody (Tonbo Bioscience) directly to the pre-incubated cell suspension in the presence of the CD16/CD32 antibody and vortex to mix.

(Note: The anti-mouse CD16/CD32 (Fc shield) antibody does not need to be washed away before staining cells with the CD11b antibody.)

10. Incubate cell suspension with the CD11b plus Fc shield antibody then mix in a 2–8 °C freezer for 30 min in the dark.

(Note: Longer incubation time, higher temperature, and incubation on ice may affect cell staining.)

11. Wash cells twice in 1 mL of FACS buffer and centrifuge at 350–400× *g* for 10 min at 20–25 °C.

(Note: Aspirate supernatant carefully, leaving around a 100 μL buffer with the cell pellet covering the bottom of the tube for the first wash, followed by complete aspiration of the supernatant in the second wash.)

12. Aspirate supernatant completely via vacuum aspiration and resuspend cell pellets (briefly vortex to dissociate the cell pellet) in a suitable amount (300–400 μL) of FACS buffer for analysis by flow cytometry (FACS) immediately, or store it in a 4 °C freezer for short-term storage. Supplemental Figure S2 shows the gating strategy and associated purity of isolated microglial populations.

(Note: Some tandem dyes like APC-Cy7/PE-Cy7 can be degraded in the presence of light, high temperature, fixation, and emitting light in the parent dye detector. Avoid the exposure of the stained sample to light, heat, and PFA based fixatives.)

### 3.7. Part 7: Proteomic Analysis (24 h)

1. Add 150 μL of RIPA extraction buffer, supplied with protease inhibitor, to microglia cell pellets from Step 49 after the 1 × DPBS wash and resuspend by pipetting up and down with a 200 μL pipette.
2. Homogenize cells on ice (to protect from overheating, proteases work best at high temperature) by sonication (total 3–6 pulse, 5-s intervals among individual pulses) to get a uniform, opaque solution with microscopic bubbles.
3. Immediately incubate the sample on ice for 10 min and then centrifuge at 15,000× *g* for 15 min at 4 °C.
4. Transfer supernatant to a fresh sterile microcentrifuge tube and keep a record of the transfer volume.
5. Quantify protein concentration by the Bradford assay or similar protein assay methods and add 3 × protein loading buffer, considering the total transfer volume.
6. Incubate the sample at 95 °C for 5 min to denature the protein, spin briefly, and use for protein loading or store at −80 °C for later use.
7. Load an equal amount of the sample (30 μg for GAPDH and IBA1 and 40 μg for GFAP and NeuN) into 10 × SDS-PAGE gel (1.5 mm thick) and run at 100 V. Load protein ladder (page ruler pre-stained protein ladder) to identify the protein band according to molecular weight.
8. Activate the PVDF membrane by pre-wetting in methanol for 10 min followed by 10 min of wetting in the transfer buffer.
9. Disassemble gel tank, carefully remove the gel, and place on the PVDF membrane, then reassemble the gel-sponge-membrane sandwich in the wet transfer tank (Bio-Rad mini protean tetra cell) containing transfer buffer and run the power supply at 300 mA for 2 h to completely transfer the protein to the PVDF membrane.
10. Disassemble the protein transfer tank, carefully remove the PVDF membrane, and block the membrane with the vacuum filtered blocking buffer (3% *w*/*v* BSA in 1 × TBST) to avoid a high background during chemiluminescence detection due to non-specific binding of the primary and secondary antibody of interest.
11. Incubate the PVDF membrane with the primary antibody of interest (diluted in 5% *w*/*v* BSA in 1 × TBST) in a GenHunter Western blot container placed on a platform shaker overnight at 4 °C (cover the membrane completely with antibody solution).
12. Remove the primary antibody after incubation, wash the membrane 3–4 times with TBST (5 min for each time), and incubate with the secondary antibody (diluted in blocking buffer) for 1 h at room temperature.
13. Remove the secondary antibody after incubation and wash the membrane 3–4 times with TBST (5 min for each time).
14. Detect the protein band by applying an equal volume of chemiluminescence solution E and F in a chemiluminescence detector (Azure biosystem, c600).

### 3.8. Parts 8 and 9: Simultaneous Purification of DNA and RNA (40 min)

(Important points before starting:

A. During the entire procedure, work quickly.
B. Perform all centrifugation steps in a standard tabletop microcentrifuge at 20–25 °C. Make sure the centrifugation chamber does not cool below 20 °C.
C. Make sure all surfaces, including benchtops, pipettors, glassware, lab coats, and solution, are RNase-free. Before starting, decontaminate all surfaces with a decontaminant, such as RNaseZap solution or RNaseZap Wipes. Always use RNase-free tubes, tips, and reagents, and change hand gloves frequently while working.
D. Make sure β-mercaptoethanol is added to the Buffer RLT Plus (lysis buffer) before use. Add 10 μL of β-mercaptoethanol per 1 mL of RLT Plus buffer.
E. Buffer RPE, AW1, and AW2 are each supplied as a concentrated solution. Add the appropriate volume of ethanol (96–100%) as indicated on each bottle to obtain a working solution before using it for the first time.)

**Figure 7:**
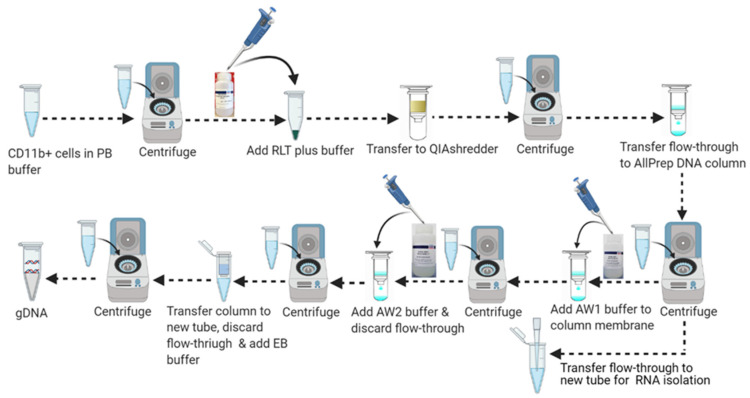
Schematic workflow for DNA purification.

1. Collect the microglial cell pellet from Step 49 and follow the DNA purification steps shown in Figure 7.
2. Loosen the cell pellet by flicking the tube 2–3 times.
3. Add 350 uL (<5 × 10^6^ cells)/600 µL (5 × 10^6^ – 1 × 10^7^) of RLT Plus buffer and mix smoothly by pipetting 8–10 times.
4. Transfer the lysate directly into a QIAshredder spin column placed on a 2 mL tube. Centrifuge at 20,000× *g* for 2–3 min.
5. Transfer the supernatant carefully (leave any solid pellet or undissolved materials at the bottom) into a Qiagen AllPrep DNA spin column. Place in a 2 mL collection tube. Close the lid carefully so that it does not touch the liquid and centrifuge at ≥8000× *g* for 30 s. Use the flow-through for RNA extraction.
6. Place the AllPrep DNA spin column in a new 2 mL blank collection tube and centrifuge at ≥8000× *g* for 30 s. After centrifugation, store the AllPrep DNA column at room temperature (15–25 °C) for immediate use or at 4 °C for later DNA purification.

(Note: Never freeze the AllPrep DNA spin column or store them on ice. Ensure that there is no liquid left on the column membrane after centrifugation; if necessary, repeat the centrifugation steps until all liquid has passed through the membrane.)

#### 3.8.1. Part 8: Genomic DNA Purification

7. Add 500 μL buffer AW1 to the AllPrep DNA spin column from step 6. Close the lid carefully so that it does not touch the flow-through and centrifuge at ≥8000× *g* for 15 s. Discard the flow-through by pipetting. Reuse the DNA spin column for the next step.
8. Add 500 μL of buffer AW2 to the AllPrep DNA spin column, close the lid carefully so that it does not touch the flow-through, and centrifuge at 20,000× *g* for 2 min to wash the DNA spin column membrane.

(Note: Remove RNeasy spin column carefully from the collection tube after centrifugation so that the column does not contact the flow-through.)

9. Place the AllPrep DNA spin column in a new 2 mL blank collection tube and discard the old collection tube with the flow-through. Centrifuge at 20,000× *g* for 1 min.
10. Place the AllPrep DNA spin column into a new 1.5 mL DNases-free collection tube. Add 15–25 μL of the appropriate volume of Buffer EB directly to the spin column membrane and close the lid carefully. Incubate at room temperature (15–25 °C) for 2–3 min followed by centrifugation at ≥8000× *g* for 1 min to elute the DNA.

#### 3.8.2. Part 9: Total RNA Purification

11. Add 1 volume or an equal volume of 70% ethanol (350–600 µL) to the flow-through in the collection tube from Step 5 and mix well by pipetting 3–5 times. Do not vortex or centrifuge. Proceed to the next step immediately.

(Note: It is important to adjust the volume of 70% ethanol with the volume of flow through left after homogenization and DNA removal steps.)

12. Pipet up to 700 µL of the sample including any precipitate from Step 11 into the RNeasy spin column and place in a 2 mL collection tube. Close the lid carefully so that it does not touch the sample and centrifuge at ≥8000× *g* for 15 s. Remove the flow-through by pipetting. Reuse the collection tube for the next step.

(Note: If the sample volume (flow-through plus 70% ethanol) exceeds 700 µL, centrifuge aliquots successively in the same RNeasy spin column. Remove the flow-through after each centrifugation.)

13. Add 700 µL buffer RW1 to the RNeasy spin column. Close the lid carefully so that it does not touch the sample and centrifuge at ≥8000× *g* for 15 s to wash the RNeasy spin column membrane. Remove the flow-through completely by pipetting. Reuse the collection tube for the next step.

[Note: Remove RNeasy spin column carefully from the collection tube after centrifugation so that the column does not contact the flow-through]

14. Add 500 µL buffer RPE to the RNeasy spin column. Close the lid carefully so that the column does not contact the flow-through and centrifuge at ≥8000× *g* for 15 s to wash the RNeasy spin column membrane. Remove the flow through completely. Reuse the collection tube for the next step.
15. Add 500 µL of buffer RPE to the RNeasy spin column. Close the lid carefully so that the column does not contact the flow-through and centrifuge at ≥8000× *g* for 2 min to further wash the spin column membrane. Remove the flow-through by pipetting. Reuse the collection tube for the next step.

(Note: After centrifugation, remove the RNeasy spin column carefully from the collection tube so that the column does not contact the flow-through.)

**Figure 8:**
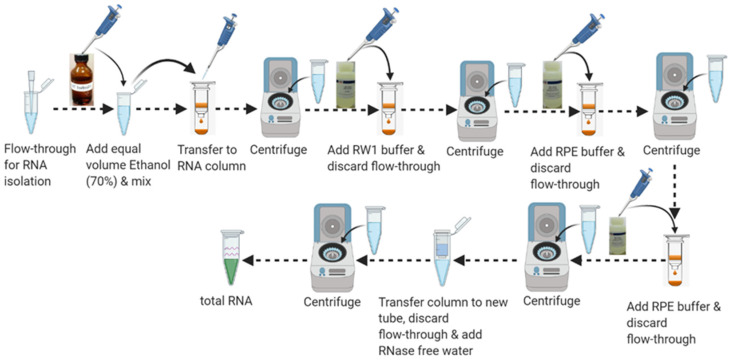
Schematic workflow for RNA purification.

16. Place the RNeasy spin column to a new 2 mL blank collection tube and discard the old collection tube with the flow-through. Centrifuge at 20,000× *g* for 1 min.
17. Place the RNeasy spin column in a new 1.5 mL RNase-free collection tube. Add 15–25 μL or an appropriate volume of RNase-free water directly to the spin column membrane. Close the lid gently and centrifuge at ≥8000× *g* for 1 min to elute the RNA as shown in Figure 8. Supplemental Figure S3 shows the quality control analysis of DNA-RNA isolated from purified microglia.

## 4. Results and Discussion

This combined method yielded purified microglia cells ranging between 3 × 10^5^ and 5 × 10^5^ cells from one adult mouse brain after double-column filtering, where the viability of purified microglia ranged between 70–80% of total cells and the purity ranged between 9 and 95%. Using Qiagen AllPrep DNA/RNA Mini kit (Step 8), total RNA yield ranged between 200 and 400 ng and genomic DNA yield ranged between 200 and 500 ng per one adult mouse brain after double-column filtering. Single-column filtering resulted in a higher number of microglial cells, ranging between 3 × 10^5^ and 6 × 10^5^, and higher cell viability, ranging between 80 and 90% of total cells, where the purity of isolated microglia was between 75 and 85%. The total RNA yield from single column filtering ranged between 250 and 600 ng and genomic DNA yield ranged between 300 ng and 1 μg. We have improved the number of purified cells from a single brain by calibrating the time and types of the brain tissue digestion system and adding an extra washing step (Step 49) for full functionality of the lysis buffer later in DNA-RNA isolation steps. Using the Qiagen AllPrep DNA/RNA/miRNA Universal kit following manufacturing instructions, DNA yield further increased to 800–1000 ng, where RNA yield increased to 500–800 ng for double-column filtering.

Purification of microglia following absolute enzymatic tissue dissociation protocol induces an abnormal gene expression signature in microglia that can mislead downstream analysis [22] and a combination of mechanical and enzymatic tissue dissociation methods was recommended very recently [23]. We used MACS^®^ Tissue Dissociation Kits with minor modifications that combine mechanical tissue dissociation with enzymatic treatment to obtain high yields of viable single cells as stated in Table 4 with preserved membrane integrity from hardly dissociated brain tissue. We used CD11b for microglia selection as it is expressed by both resting and activated microglia. Although, some recent studies [24,25] suggested TMEM119 as a more specific marker that can distinguish microglia from infiltrating macrophages in pathological condition. However, TMEM119 expression is absent in immature microglia [25] and changes in immature microglia are critical for downstream studies, as it can lead to persistent changes in microglial function, resulting in long-term neuronal dysfunction [26]. Moreover, sorting of microglia (FACS) with TMEM119 selection from a single mouse brain yields a smaller number of viable cells compared to MACS CD11b selection [27].

**Figure 9:**
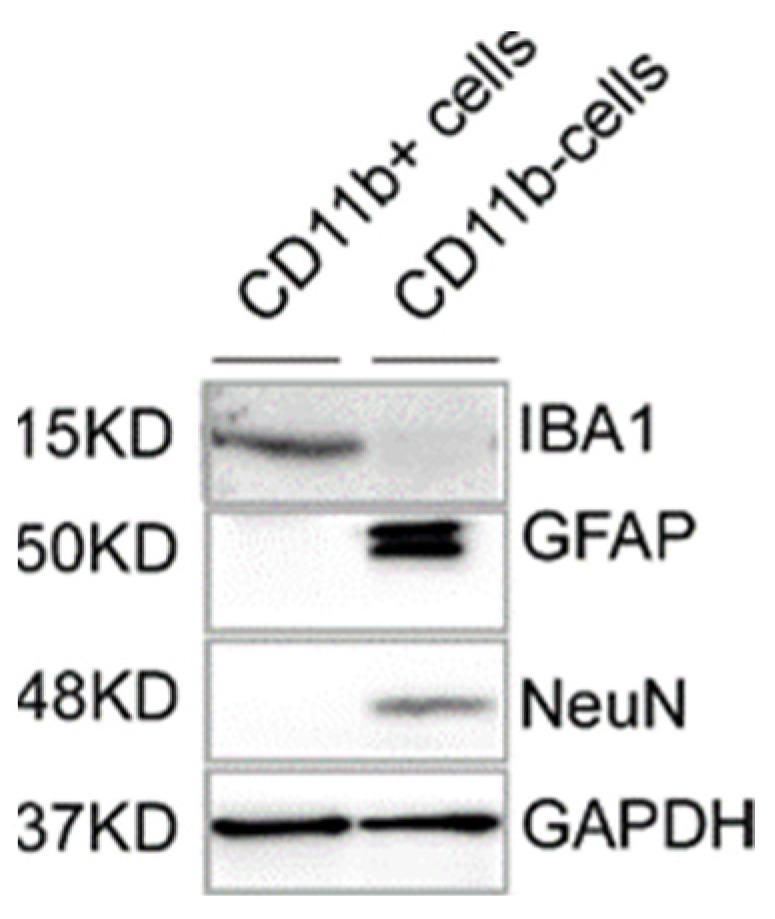
Purity of isolated mouse microglia population by proteomic analysis. Cell lysates from the CD11b positive and negative fraction were subjected to Immunoblotting. Protein band captured in immunoblot analysis after probing with GAPDH (loading control), GFAP (astrocyte), IBA1 (microglia), and NeuN (Neurons) antibody. CD11b-positive cell lysates were positive for IBA1 but negative for GFAP and NeuN, whereas CD11b-negative cell lysates were negative for IBA1 but positive for GFAP and NeuN.

This method yielded 500–800 μg of protein from one adult mouse brain and overcame the BSA contamination issue as evident by the immunoblotting images in Figure 9. BSA contamination in the extracted protein sample was minimized by a single 1 × DPBS wash (Step 49). Although we used a MACS enzymes system for tissue dissociation steps, we also tested a Collagenase-Dispase-DNaseI system that yields similar results. We have successfully used this method for microglia DNA-RNA isolation from a single, whole brain of an adult mouse. We believe it is possible to isolate microglia and microglial DNA-RNA from specific brain regions using this method, but optimization of the method, especially tissue dissociation and enzyme digestion, are highly recommended before use.

## 5. Conclusions

Our current method describes details of an efficient DNA-RNA isolation method from purified microglia from one adult mouse brain suitable for microglial transcriptomics and proteomics analysis. This method can also be used for any study requiring microglial single-cell transcriptomic profiling, DNA methylation analysis, proteomics analysis by Western blot, qRT-PCR, and immunophenotyping by flow cytometry in any number of adult mice.

## Figures and Tables

**Table 1:** Detailed information of materials.

Name	Source	Identifier	Location
**Chemical, kit, and supplies**			
MACS adult mouse brain dissociation kit	Miltenyi Biotec	130-107-677 (O.N)	Auburn, CA, USA
DNase I (1 U/μL)	Invitrogen	55803	Eugene, OR, USA
PageRuler^TM^ Prestained Protein ladder (26616)	Thermo Fisher Scientific	815-968-0747	Waltham, MA, USA
Tetramethylethylenediamine (TEMED)	Thermo Fisher Scientific	KA1180732	Waltham, MA, USA
Sodium Dodecyl Sulfate (SDS)	Thermo Fisher Scientific	BP166-500	Waltham, MA, USA
NaOH pellet	Thermo Fisher Scientific	BP359-212	Waltham, MA, USA
Protease inhibitor cocktail	Thermo Fisher Scientific	NE1509703	Waltham, MA, USA
Pentobarbital (1 mg/mL in 1 mL methanol)	Cerilliant Corporation	P-010-1ML	Round Rock, TX, USA
1× PIERCE RIPA buffer	Thermo Fisher Scientific	89900s	Waltham, MA, USA
PVDF transfer membrane (0.45 μm), Immobilon	Bio-Rad	IPVH00010	Hercules, CA, USA
Tween 20	Acros Organics	A015198101	Geel, Antwerp, BE
Tris base	Thermo Fisher Scientific	BP 152-5	Waltham, MA, USA
Glycine	Thermo Fisher Scientific	BP381-5	Waltham, MA, USA
Sodium Chloride (NaCl)	Thermo Fisher Scientific	S671-10	Waltham, MA, USA
30× Acrylamide (Protogel)	National Diagnostics	EC-890	Atlanta, GA, USA
1× Penicillin/streptomycin	Thermo Fisher Scientific	15140122	Waltham, MA, USA
Bovine serum albumin (BSA) solid	Thermo Fisher Scientific	BP1600-100	Waltham, MA, USAS
Ethylenediaminetetraacetic acid (EDTA)	Millipore Sigma	58F-0292	Burlington, MA, USA
CD11b (microglia) Microbeads, human and mouse	Miltenyi Biotec	130-093-634 (O.N)	Auburn, CA, USA
RNase & DNase free tips (250 µL)	Rainin	0703BLTS	Oakland, CA, USA
RNase & DNase free tips (20 µL)	CELLTREAT	229017	Pepperell, MA, USA
RNase & DNase free tips (1 mL)	Thermo Fisher Scientific	02707402	Waltham, MA, USA
RNase away wipes	Molecular Biology Products	7007	Toronto, Ontario, CA
Polystyrene round bottom tube (5 mL)	Becton Dickinson Labware	130-091598	Franklin Lakes, NJ, USA
Syringe filter (0.2 μm)	Nalgene	190-2520	Rochester, NY, USA
Syringe (5 mL), sterile	Becton Dickinson Labware	309646	Franklin Lakes, NJ, USA
Conical tube (15 & 50 mL)	Sarstedt, Inc	62554.205 & 62547.100	Newton, NC, USA
Microcentrifuge tube (1.5 mL), DNase & RNase free	Thermo Fisher Scientific	AM12400	Waltham, MA, USA
AllPrep DNA/RNA mini kit	Qiagen	160014919	Hilden, Germany
AllPrep DNA/RNA/miRNA Universal Kit	Qiagen	166047328	Hilden, Germany
2-mercaptoethanol (β-ME)	Thermo Fisher Scientific	21985-023	Waltham, MA, USA
Ammonium persulfate (APS)	National Diagnostics	National Diagnostics, EC-504	Atlanta, GA, USA
Glutamic acid	Millipore Sigma	G7029	Burlington, MA, USA
MACS pre-separation filter (70 μm & 30 μm)	Miltenyi Biotec	130-095-823 (O.N)	Auburn, CA, USA
QIAshredder (REF79654)	Qiagen	160010887	Hilden, Germany
MACS separation column (LS)	Miltenyi Biotec	130-042-401	Auburn, CA, USA
Disposable vacuum filter, 0.22 µm (50 mL)	Millipoe Sigma	SCGP00525	Burlington, MA, USA
Cell culture petri dish (60 × 15 mm)	Becton Dickinson Labware	35-3002	Franklin Lakes, NJ, USA
Countess cell counting chamber slide	Invitrogen	2C11940P	Eugene, OR, USA
**Antibody and dyes**			
Anti-mouse GFAP antibody	Millipore Sigma	G3893	Burlington, MA, USA
Anti-rabbit IBA1 antibody	Wako	016-20001	Chuo-ku, Osaka, JP
Anti-mouse GAPDH antibody	Santa Cruz Biotechnology	sc32233	Dallas, TX, USA
Anti-rabbit NeuN antibody	Invitrogen	PA578499	Eugene, OR, USA
Anti-mouse IgG-HRP, secondary antibody	Santa Cruz Biotechnology	sc516102	Dallas, TX, USA
Anti-rabbit IgG-HRP, secondary antibody	Santa Cruz Biotechnology	sc2317	Dallas, TX, USA
Anti-mouse/-human FITC-CD11b antibody M1/70	Tonbo Biosciences	35-0112-U100	San Diego, CA, USA
Anti-mouse CD16/CD32 (Fc shield) antibody 2.4G2	Tonbo Biosciences	70-0161-U500	San Diego, CA, USA
Ghost Dye violet 510 (viability dye)	Tonbo Biosciences	13-0870-T100	San Diego, CA, USA
Trypan blue stain (0.4×) solution	Thermo Fisher Scientific	15250-061	Waltham, MA, USA
**Ready stock solution**			
DPBS (with calcium/magnesium)	Thermo Fisher Scientific	14040-133	Waltham, MA, USA
DPBS (without calcium/magnesium)	Hyclone Laboratories, Inc	SH30028.02	Logan, UT, USA
HBSS (with calcium/magnesium)	Thermo Fisher Scientific	14025-092	Waltham, MA, USA
HBSS (without calcium/magnesium)	ATCC	30-2213	Manassas, VA, USA
Distilled Water	Thermo Fisher Scientific	15230-162	Waltham, MA, USA
RNase free surface decontaminant	APExBIO	10-228	Houston, TX, USA
MACS BSA stock solution	Miltenyi Biotec	130-091-376 (O.N)	Auburn, CA, USA
HyPure molecular biology grade water, Hyclone	Genesee Scientific	87-1162	El Cajon, CA, USA
MACS debris removal solution	Miltenyi Biotec	130-107-677 (O.N)	Auburn, CA, USA
MACS red blood cell (RBC) removal solution	Miltenyi Biotec	130-107-677 (O.N)	Auburn, CA, USA
Diethyl pyrocarbonate (DEPC)	RPI	D43060-25.0	Mount Prospect, IL, USA
Dulbecco’s Modified Eagle Medium (DMEM)	ATCC	30-2002	Manassas, VA, USA
Pierce Chemiluminescence solution (ECL solution)	Thermo Fisher Scientific	89880E & F	Waltham, MA, USA
RNase free water	Thermo Fisher Scientific	BP561-1	Waltham, MA, USA
Fetal bovine serum (FBS) liquid	R&D Systems	S11150H	Minneapolis, MN, USA
Methanol	Thermo Fisher Scientific	A412-4	Waltham, MA, USA
Ethanol (absolute), 200 proof	Thomas Scientific	05K16PA	
**Experimental animal strain**			
Male, C67BL6J mice, 7 months old, body weight 46.23 g	University of North Dakota	n/a	
Male, Thy1-aSyn mice, 7 months old, body weight 39 g	University of North Dakota	n/a	
**Others**			
GraphPad Prism	GraphPad Software	n/a	
ImageJ	National Institute of Mental Health	Imajej.nih.gov/ij/download.html	
BioRender	BioRender	n/a	

**Table 2:** Detailed information of the equipments.

Name	Supplier/Manufacturer	Location
Refrigerated Ultracentrifuge	Eppendorf, model 5417R	Enfield, CT, USA
Refrigerated Rotor with buckets	Sorvall-RT7 Plus	Hampton, NH, USA
Glass Pasteur Pipet (sterile)	Thermo Fisher Scientific	Waltham, MA, USA
Scissors and knife (sterile)	FEATHER safety razor	Kita-Ku, Osaka, JP
MACS MultiStand	Miltenyi Biotec	Auburn, CA, USA
MACS Separation Columns (MS)	Miltenyi Biotec	Auburn, CA, USA
MACS magnet for MS column	Miltenyi Biotec,	Auburn, CA, USA
−80° freezer	Thermo Fisher Scientific	Waltham, MA, USA
Reciprocal shaking water bath	Precision Scientific, model 25	Winchester, VA, USA
4–8° freezer	Biomedical Solutions, Inc	Stafford, TX, USA
Speed Vacuum System (SC200)	Savant Systems Inc	Hyannis, MA, USA
End Over End shaker	Barnstead Thermolyne Corporation	Ramsey, MN, USA
CO_2_ water-jacketed incubator	NUAIRE IR Autoflow	Plymouth, MN, USA
Countess II automated cell counter	Life Technologies	Carlsbad, CA, USA
Chemiluminescence detector system	Azure biosystem, c600	Dublin, CA, USA
Vortex mixer	Thermo Fisher Scientific, 02215365	Waltham, MA, USA
Gel electrophoresis power supply	Bio-Rad, 041BR85979	Hercules, CA, USA

**Table 3:** Detailed recipe of the freshly prepared solutions.

Name	Recipe
PB buffer	Dissolve 0.5 × BSA in 1 × DPBS, sterile filter, degas, and cool to 2–4 °C
1 × PBS	Mix 8 g NaCl, 0.2 g KCl, 1.15 g Na_2_HPO_4_, 0.24 g KH_2_PO_4_ in 800 mL H_2_O, adjust pH to 7.4, add H_2_O until volume 1L
FACS buffer	Dissolve 2%FBS in 1 × DPBS, add 5 mM EDTA, sterile filter
FACS stain buffer	Dissolve 2%FBS in 1 × DPBS, sterile filter
MACS Enzyme Mix-I (EM-1)	Mix 1900 μL Buffer Z and 50 μL Enzyme P (prepare fresh)
MACS Enzyme Mix-II (EM-2)	Mix 20 μL Buffer Y and 10 μL Enzyme A
500 mM EDTA solution, pH 8.0	Mix 186.1 g Na_2_-EDTA in 800 mL dH_2_O, adjust pH 8.0 by adding NaOH pellets and make volume 1 L with dH_2_O
Transfer Buffer	Mix 14.4 g glycine and 3 g Tris in 800 mL dH_2_O, add 200 mL methanol to make the volume 1 L
Running Buffer	Mix 14.4 g glycine, 3 g Tris and 1 g SDS in 800 mL dH_2_O, adjust pH to 8.3 and add dH_2_O till the volume 1 L
Stacking gel (4×)	dH_2_O 2.7 mL, 30×acrylamide 0.67 mL, 1.0 mol/L Tris (pH 6.8) 0.5 mL, 10%SDS 40 µL, 10%APS 40 µL and TEMED 4.0 µL
Resolving gel (10× and 15×)	dH_2_O 4 mL, 30 × acrylamide 3.3 mL, 1.5 mol/L Tris (pH 8.8) 2.5 mL, 10%SDS 100 µL, 10%APS 100 µL and TEMED 4.0 µL (10×) dH_2_O 2.3 mL, 30 × acrylamide 5 mL, 1.5 mol/L Tris (pH 8.8) 2.5 mL, 10%SDS 100 µL, 10%APS 100 µL and TEMED 4.0 µL (15×)
Washing buffer (1 × TBST)	Mix 8.0 g NaCl, 0.2 g KCl and 3.0 g tris base in 800 mL dH_2_O, add 0.1% tween20, adjust pH 8.0, then add dH_2_O till the volume 1 L, autoclave and store at room temperature (RT)
Blocking buffer	Dissolve 3% BSA in 1 × TBST, sterile filter

**Table 4:** Expected yields. This table summarizes the yield of microglia and subsequent DNA/RNA isolation using both single and double column filtering as well as the Qiagen mini and universal kits. The ranges represent the minimum and maximum results obtained from eight seven-month-old mice.

Filtering	CD11b+ Cells	Viability (%)	Purity (%)	DNA (mini)	RNA (mini)	DNA (Universal)	RNA (Universal)
Single Filter	(3.3–6.0) × 10^5^	85–91%	73–78%	300–1100 ng	250–625 ng	1000–1505 ng	900–1000 ng
Double Filter	(3.0–5.1) × 10^5^	71–80%	90–95%	200–510 ng	200–410 ng	780–1050 ng	750–800 ng

## Data Availability

The data presented in this study are available within the figures and supplemental material.

## Supplementary Materials

The following are available online at https://www.mdpi.com/article/10.3390/mps4040086/s1. Figure S1, Pre & post centrifugation gradients in debris removal step. DPBS: Dulbecco’s Phosphate Buffered Saline, Figure S2, Cell gating strategy and gating images in FACS analysis. Purified microglial cells were labeled with APC-CD11b, and viability dye (violet 510, Tonbo Biosciences). (A) Representative images for all cells gating. (B) Corresponding singlet cell gating image after gating from (A). (C) Representative gating on histogram plot for live cells. (D) Representative gating on histogram plot for CD11b positive cell after gating from (C), Figure S3, Quality control analysis of isolated DNA-RNA from purified microglia cells. (a) Total RNA was isolated from purified microglia cells using the AllPrep DNA/RNA Mini Kit, RNA band intensities from wild type and transgenic mice (2) captured by RNA gel electrophoresis, corresponding RNA integration value, RIN calculated by peak area measurement. (b) Genomic DNA was isolated from purified microglia cells using the AllPrep DNA/RNA Mini Kit, genomic DNA band intensities from wild type and transgenic mice (2) captured by DNA gel electrophoresis, corresponding DNA integration value, DIN calculated by peak area measurement.
