# Supplementary material for: Method of Microglial DNA-RNA Purification from a Single Brain of an Adult Mouse
Source: Methods Protoc. 2021 Dec 2;4(4):86. doi: 10.3390/mps4040086 (PMC8704779; doi:10.3390/mps4040086)
Supplement: Supplementary file 1 [file mps-04-00086-s001.zip › mps-1447412-supplementary.pdf]

Supplemental Information

Method of Microglial DNA-RNA purification from Adult Mouse Single Brain

Md. Obayed Raihan, Brett A. McGregor, Nathan A. Valeris, Afrina Brishti, Junguk Hur, and James E. Porter

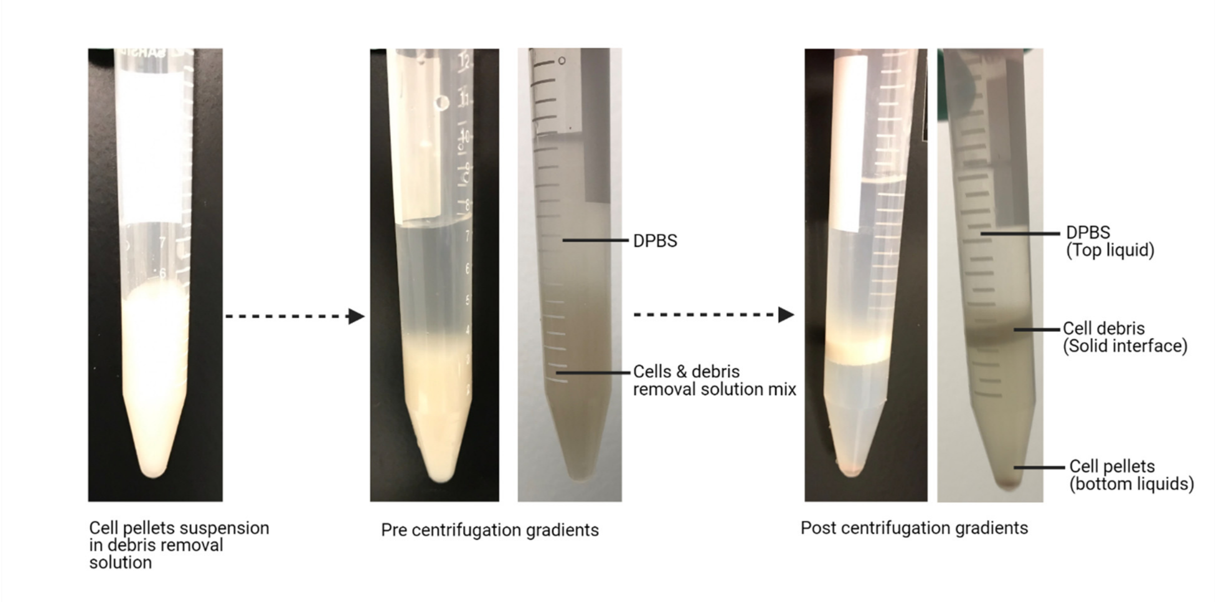

Figure S1. Pre & post centrifugation gradients in debris removal step. DPBS: Dulbecco's Phosphate Buffered Saline.

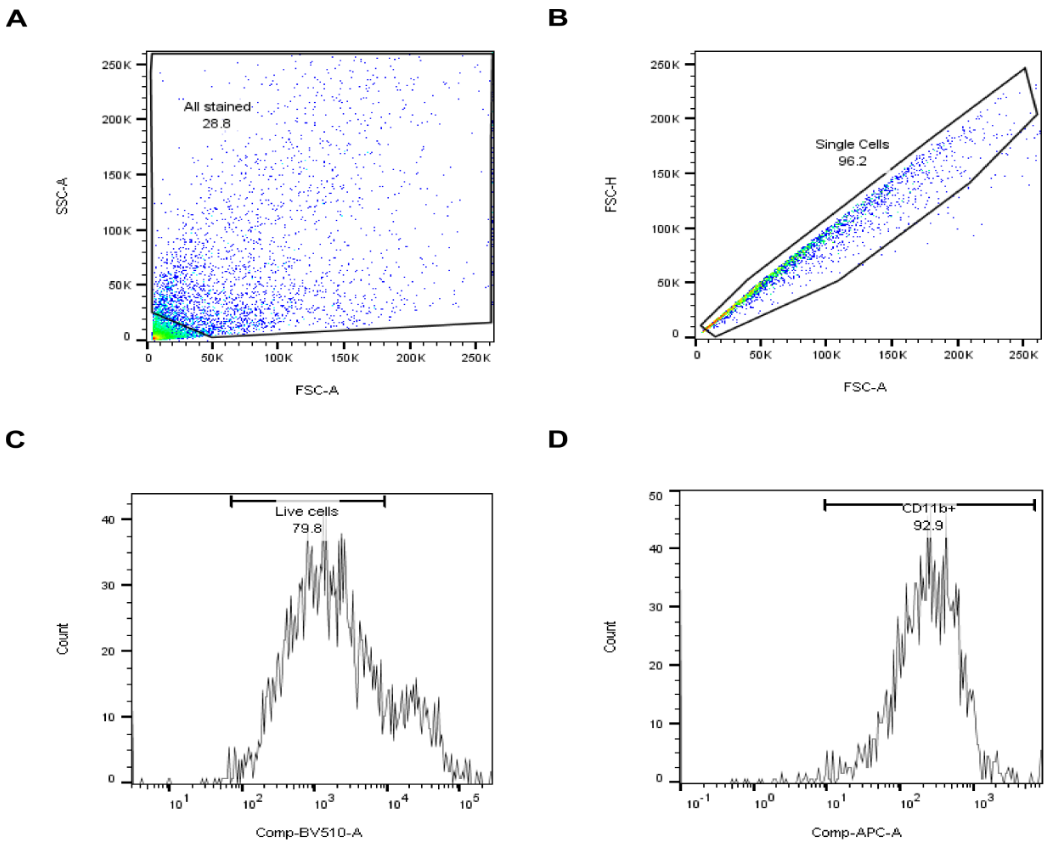

Figure S2. Cell gating strategy and gating images in FACS analysis. Purified microglial cells were labeled with APC-CD11b, and viability dye (violet 510, Tonbo Biosciences). (A) Representative images for all cells gating. (B) Corresponding singlet cell gating image after gating from (A). (C) Representative gating on histogram plot for live cells. (D) Representative gating on histogram plot for CD11b positive cell after gating from (C).

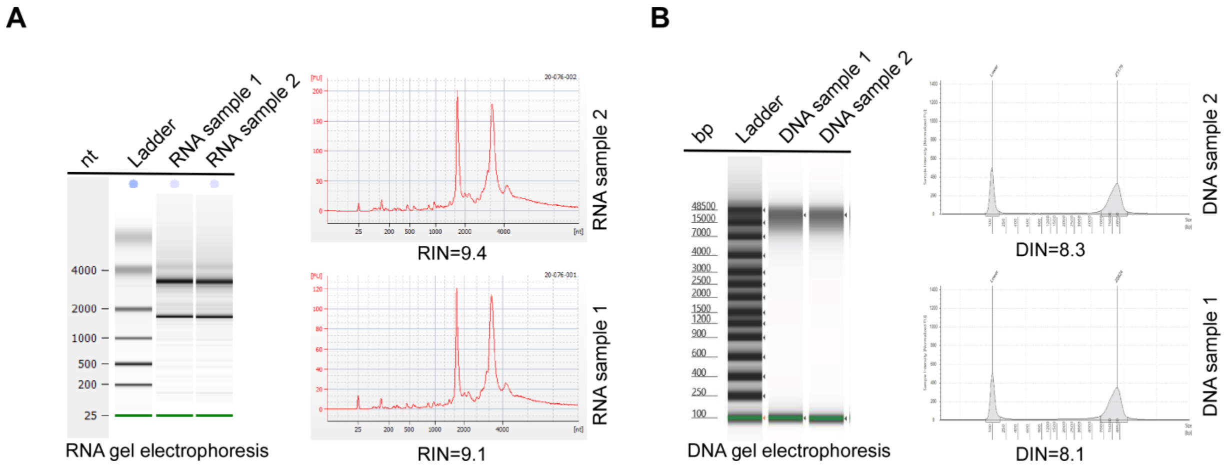

Figure S3. Quality control analysis of isolated DNA-RNA from purified microglia cells. (a) Total RNA was isolated from purified microglia cells using the AllPrep DNA/RNA Mini Kit, RNA band intensities from wild type and transgenic mice (2) captured by RNA gel electrophoresis, corresponding RNA integration value, RIN calculated by peak area measurement. (b) Genomic DNA was isolated from purified microglia cells using the AllPrep DNA/RNA Mini Kit, genomic DNA band intensities from wild type and transgenic mice (2) captured by DNA gel electrophoresis, corresponding DNA integration value, DIN calculated by peak area measurement.
